# Release Our Hostages Now!

**DOI:** 10.5041/RMMJ.10507

**Published:** 2023-10-29

**Authors:** Itamar Ashkenazi, Rafael Beyar, Shraga Blazer, Aaron Ciechanover, Karl L. Skorecki

**Affiliations:** 1 Editor, Rambam Maimonides Medical Journal; 2 Attending Physician, Department of General Surgery, Rambam Health Care Campus, Haifa, Israel; 3 Ruth and Bruce Rappaport Faculty of Medicine, Technion–Israel Institute of Technology, Haifa, Israel; 4 Interventional Cardiology, Rambam Health Care Campus, Haifa, Israel; 5 Editor in Chief, Rambam Maimonides Medical Journal; 6 Rambam Health Care Campus, Haifa, Israel; 7 Nobel Laureate in Chemistry (2004); 8 Former Dean, Azrieli Faculty of Medicine, Bar-Ilan University, Safed, Israel

**Keywords:** Hamas, hostages, Israel, medical ethics, terrorism

It has been the policy of *Rambam Maimonides Medical Journal* to limit the number of editorials published. However, silence and standing on the sidelines is not an option in light of the atrocities and inhumanity we witnessed on October 7. The savagery of the Hamas massacre was executed indiscriminately upon children, women, older people (some of whom are Holocaust survivors), infants, and even medical professionals caring for the casualties. Currently, there are about 230 women, men, children, and babies being held hostage by Hamas; among them are cancer patients and others with serious disorders, doctors, and other medical professionals. We cannot rest and must address the plight of our hostages who are being held by terrorists motivated by hatred and showing no respect for life, whether that of their enemies, their own people, or even themselves.

For many years, the international community has criticized Israel for positioning itself against the Palestinian aspirations for autonomy. Yet, for the past 20 years, hardly any criticism has been directed at Hamas and the Islamic Jihad for their indiscriminate firing of thousands of rockets and mortars upon Israel’s citizens. Moreover, the international community has largely remained silent about the intentional and indiscriminate murder of innocent civilians through bombings, shootings, and stabbings perpetrated by individuals claiming to be “freedom fighters.” Barely a word is spoken—or published—about the abducted Israeli civilians and soldiers who, for years, remain hostages of Hamas. They are secluded from civilization with no fundamental rights guaranteed by international law.

We call upon all members of the global medical community to demand the immediate release of all the hostages abducted on October 7. Furthermore, we call upon the global medical community to condemn anyone who supports Hamas, Hezbollah, the Islamic Jihad, and every other form of terrorism, and demand the immediate disarmament and dismantling of these organizations. Organizations whose main purpose is to destroy those who differ from them—whatever their rationale—do not merit the empathy of those who cherish life. Their moral values have nothing in common with those that the medical profession stands for, and for which all too many medical professionals have died.

The international medical community was quick to accuse Israel of bombing the al-Ahli Hospital in Gaza before all the facts had become available. It did not take long to provide incontrovertible evidence that this disaster resulted from a failed missile launched by the Islamic Jihad, targeted at Israeli civilians. How is it that so many leaders, representatives, and members of the medical and academic community, which prides itself on evidence-based conclusions, rushed to condemn Israel based on Hamas’s false and libelous reports that Israel had targeted the al-Ahli Hospital? We are painfully aware of the deadly consequences of promulgating false accusations against Jews throughout history. Additionally, we expect the leaders of medicine and academia to be able to distinguish between the sad reality of injury and death of untargeted non-combatants, and the deliberate singling out of civilians (Arab and Jewish) by Hamas as was done on October 7, 2023. The horrors committed by Hamas include the sacrificial death of their own in the form of human shields on the one hand, while, on the other hand, specifically instructing the terrorists as to which civilians to gruesomely murder, torture, rape, mutilate, and kidnap. Numerous documents surrendered by the captured terrorists give testimony that these horrors were committed under the directives of the Hamas leadership and fueled by Captagon pills.

We should all be truthful about the casualties of a war forced upon Israel and initiated by a terror organization with the stated aim of exterminating the Jewish state and its supporters.^1^ To that end, they use their own schools to launch rockets, their prayer houses as ammunition depots, their hospitals as headquarters, and their own people as human shields. The consequences are dire when highly respected medical and academic leaders confuse their students, protégées, and each other by making cowardly and ambiguous statements that fail to distinguish between Israel and Hamas and other terror organizations. They become actively complicit in the perpetuation of hostilities that are spilling over in university campuses including faculties of medicine and medical centers worldwide, where Jewish people are now being threatened and harassed. When students chant on-campus, “from the river to the sea, Palestine will be free,” the presidents, provosts, deans, and medical center directors should know that this chant is a call for exterminating the more than seven million Jews residing in Israel—not one Jew remaining on any swath of the land of sovereign Israel—in short, no Jewish State.

Silence is complicity. Israeli hostages in Gaza must not be added to the gruesome toll of the dead and maimed resulting from the pogrom of October 7, 2023, when 1,400 Israelis were exterminated and many more injured.

The World Health Organization recently called on Hamas to provide proof that the abductees are alive, and to release all of them. The Director-General of the World Health Organization, Tedros Adhanom Ghebreyesus, said: “There is an urgent need for the captors of the hostages to provide signs of life, proof of provision of health care and the immediate release, on humanitarian and health grounds, of all those abducted.”^2^

Our mandate and privilege as Israeli medical professionals is to do good even in wartime. We are obliged to aid the injured, whether friend or foe. We have shared knowledge with medical professionals for the benefit of the Palestinian population. Furthermore, we continue to treat children from Gaza and the Palestinian Authority with cancer and other critical conditions here in Israel, despite the current hostilities. We had hoped to be the bridge to future peace. The October 7 massacre has shattered that trust. Do not be complicit in murderous fabrications and lies; join in our demand to immediately and unconditionally release all the hostages. Do it now!
